# Medication-overuse headache: a narrative review

**DOI:** 10.1186/s10194-024-01755-w

**Published:** 2024-05-31

**Authors:** Helin Gosalia, David Moreno-Ajona, Peter J. Goadsby

**Affiliations:** 1 NIHR King’s Clinical Research Facility, & SLaM Biomedical Research Centre, The Wolfson Sensory, Pain and Regeneration Centre (SPaRC), Institute of Psychiatry, Psychology and Neuroscience (IoPPN), King’s College London, London, UK; 2 Department of Neurology, Queen Elizabeth Hospital, London, UK; 3 Department of Neurology, University of California, Los Angeles, CA USA; 4 Wellcome Foundation Building, King’s College Hospital, London, SE5 9PJ UK

**Keywords:** Medication overuse, Acute, Migraine, Preventive, Chronic migraine, Withdrawal

## Abstract

Medication-overuse headache (MOH), which potentially involves 1–2% of the population, is defined as a headache, on ≥ 15 days a month affected, along with overuse of one or other acute attack medications. MOH presents with significant challenges in the headache community, particularly in clinical settings raising various questions about its pathophysiology. Through a review of the current literature and our clinical experience, we have explored the mechanisms through which MOH may occur, provide an understanding of the current state of treatment and detail some possible views on the understanding and treatment of this condition. We evaluate the variations in treatment methods offered globally and understanding of the disorder. Above all interventions, patient education is crucial, which is underscored by an analysis of the academic publications. Given the condition is preventable, early intervention is imperative and patient awareness is highlighted as key. Globally, there is no uniform treatment methodology, which may be advantageous as approaches need to take local circumstances into account.

## Introduction

Medication-overuse headache (MOH) is a well-established cause of chronic daily headache: a term applied for patients with 15 or more headache days a month for more than 3 months [1]. It is estimated that MOH prevalence is 1–2% among the general population, and it can reach up to 50% among patients with chronic headache [2], as well as up to 50% of patients attending specialised tertiary headache centres [3]. In this review, we set out to discuss and critically evaluate MOH, highlighting key issues and aspects of this subject for the medical and scientific community. Through reviewing and synthesising published literature via ‘PubMed’ and ‘Google Scholar’, the objective is to offer the field an updated view of MOH in the context of our clinical experience.

## MOH in the population

Patients with chronic (frequent) headache, either migraine or tension-type headache, were first reported to overuse ergotamine as a pain killer with withdrawal of this substance led to headache amelioration in 1951 [4]. The same authors reported 52 migraine patients with excessive use of ergotamine preparations, which also included caffeine, barbiturates as well as Belladonna alkaloids in some cases [5]. It was not until the 1980s, however, that overuse of drug combinations including acetylsalicylic acid, codeine, ergotamine, caffeine, and barbiturates among others, was related to an increase in headache which improved after withdrawal of these substances [6]. A more recent longitudinal study revealed that patients with episodic migraine were more likely to progress to chronic migraine if they were taking medication containing triptans [7, 8], opioids or barbiturates at relatively high frequencies, suggesting a medication-dependent effect [9]. These results have been replicated in other studies, and triptans and ergots, among other analgesics, have also been shown to cause headache progression with differences in time required for withdrawal [10].

The current International Classification of Headache Disorders-3 (ICHD-3) includes MOH as a separate secondary disorder, described as headache occurring on 15 or more days/month for at least three months in a patient with a pre-existing headache disorder as a consequence of overuse of acute symptomatic medication [11] (Table 1).

**Table 1:** Acute medication usage thresholds for medication overuse headache [11]

Drug class	Threshold for medication overuse headache
Ergotamine	≥ 10 days per month for more than 3 months
Triptan	≥ 10 days per month for more than 3 months
Non-steroidal anti-inflammatory drugs (NSAID)	≥ 15 days per month for more than 3 months
Paracetamol (acetaminophen)	≥ 15 days per month for more than 3 months
Opioids	≥ 10 days per month for more than 3 months
Combination analgesics	≥ 10 days per month for more than 3 months
Multiple drug classes	≥ 10 days per month for more than 3 months

Currently, MOH is a well-accepted cause of chronic daily headache [12]. The International Classification of Headache Disorders 3rd edition (ICHD-3) does not require a demonstration of causality to establish the diagnosis in contrast with previous classifications. This is practical in that it makes the classification simpler and easier to use. However, the possibility that some of what is “overuse” is simply a consequence of severe and frequent headache, i.e. driven the underlying disease, needs to be considered. Nevertheless, in terms of establishing the treatment and prognosis of this presumable secondary headache, it opens the question of who has a real secondary headache because of the use of painkillers, who has a headache that does not change regardless of the medications taken, and who is more at risk of developing MOH.

Additionally, the classification does partly take into account that the evidence for the development of MOH is not the same for all acute medications or their combinations. Overuse of simple analgesics is considered above 15 days a month, whereas 10 or more days taking opioids or triptans as well as combinations of painkillers, are defined as MOH. Indeed, a longitudinal study revealed that patients with episodic migraine were more likely to progress to chronic migraine if they were taking medication containing opioids or barbiturates, suggesting a medication-dependent effect [9]. These results have been replicated in other studies and triptans, and ergots among other analgesics have also been shown to cause headache progression with differences in time required for withdrawal [10].

Indeed, are some patients simply responding to increased attack frequency by increasing acute attack medication use? Certainly, there is evidence supporting a view that migrainous biology is a predisposing factor [13]. A study of 110 rheumatology patients showed that 8 of them had chronic daily headache, which were preceded by regular use of analgesics in 5/8 patients. Interestingly, every patient with MOH had a previous history of migraine suggesting a predisposition of patients with a pre-existing primary headache to develop MOH [14]. Conversely, a similar study in patients attending a Rheumatology clinic demonstrated a very low prevalence of migraine (< 2%) among patients who developed chronic daily headache, although 32% were said to have regular headache [15]. The authors concluded that doctors should have greater awareness of the potential risk of developing a secondary headache if excessive analgesia was used. A study of Wilkinson and colleagues explored whether overuse of opiates (sic.) induces chronic daily headache in those with migraine who were post colectomy for ulcerative colitis for at least 1 year prior. Through a questionnaire, data were collected regarding chronic daily headache and questions related to their prior surgery. The results indicated that 34% of the returned questionnaire cohort had a history of migraine. Eight of the patients in the cohort identified as exceeding the limit for opiate use in patients with headache. The authors concluded that those who used daily opiates to control bowel motility following surgery developed chronic daily headache post-surgery [16].

The development of gepants, CGRP receptor antagonists, for the preventive treatment of migraine offers an important advance since if these medicines, useful also for acute treatment do not cause MOH, this would be a crucial advance. Laboratory pre-clinical data show that chronic administration of either olcegepant [17] or ubrogepant [18] does not, in contrast to a triptan, produce sensitisation. When given daily, atogepant [19–21], or every other day, rimegepant [22], both gepants are more effective than placebo as migraine preventives. At an individual analysis level, more participants had increased headache frequency with placebo than with atogepant in both its episodic and chronic migraine studies [23].

## MOH biology

The biological explanation for the development of MOH is still under debate. The pathophysiology of this entity is not well understood, despite some structural and functional neuroimaging studies involving MOH patients. To what extent migraine and MOH have possible shared pathophysiology is yet to be elucidated.

Structurally, a voxel-based morphometry brain MRI study showed an increase in grey matter volume of different brain areas (midbrain, thalamus and striatum) with a decrease in frontal regions [24]. Namely, the midbrain grey matter changes including the periaqueductal grey (PAG) seemed to resolve after detoxification [25]. In the same study, decreased grey matter in the orbitofrontal cortex was predictive of a poor response to treatment [25]. Similarly, greater grey matter volume in the orbitofrontal cortex has been shown to predict a better response to medication overuse treatment [26].

From a metabolic point of view, a fluorodeoxyglucose - positron emission tomography (^18^FDG PET) study in 16 chronic migraine patients with medication overuse, before and 3 weeks after medication withdrawal, showed hypometabolism in the thalamus, orbitofrontal cortex, anterior cingulate gyrus, insula/ventral striatum and right inferior parietal lobule and hypermetabolism in the cerebellar vermis, all of which resolved after withdrawal except for the hypoactivity in the orbitofrontal cortex [27]. In functional magnetic resonance imaging (MRI) studies, the lateral pathway of the pain matrix, along with the somatosensory cortex, inferior parietal lobe and supramarginal gyrus have been demonstrated to have reduced activations in MOH patients. These changes were reversible if medication overuse was stopped [28, 29]. In fact, a fMRI study performed while stimulating patients with mechanical pain showed less activation of the lateral pathway of the pain matrix in chronic migraine-MOH patients compared to controls [30].

The mesocorticolimbic dopamine circuit, which is known to be altered in addiction, was studied in MOH patients by focusing on the ventromedial prefrontal cortex and substantia nigra/ ventral tegmental area complex, showing a dysfunction compared to controls and chronic migraine patients without medication overuse [31]. The authors suggested that MOH may share some neurophysiological features with addiction. This has been investigated further in a resting state study, and the functional connectivity of the nucleus accumbens and dorsal rostral putamen has been shown to discriminate MOH and non-MOH patients with 75% and 66% accuracies, respectively [32]. Another important structure in episodic memory and awareness, the precuneus, is altered in MOH patients compared to episodic migraine patients and healthy controls [33].

Only one study has compared MOH with a different type of pain (chronic myofascial pain) by measuring resting state and diffusion tensor imaging. This showed hyperconnectivity of the salience network with abnormal connectivity between the PAG and frontal regions in the MOH group [34].

No definite evidence has been found to explain fully the biology of medication overuse headache. Beyond the aforementioned neuroimaging studies, we propose the development of medication overuse headache may be a migraine-related phenomenon. Indeed, a study on Rheumatology patients who typically take regular painkillers showed how only those with a previous history of migraine developed medication overuse headache when exposed to acute medications [14].

## Therapies for MOH

**Fig. 1:**
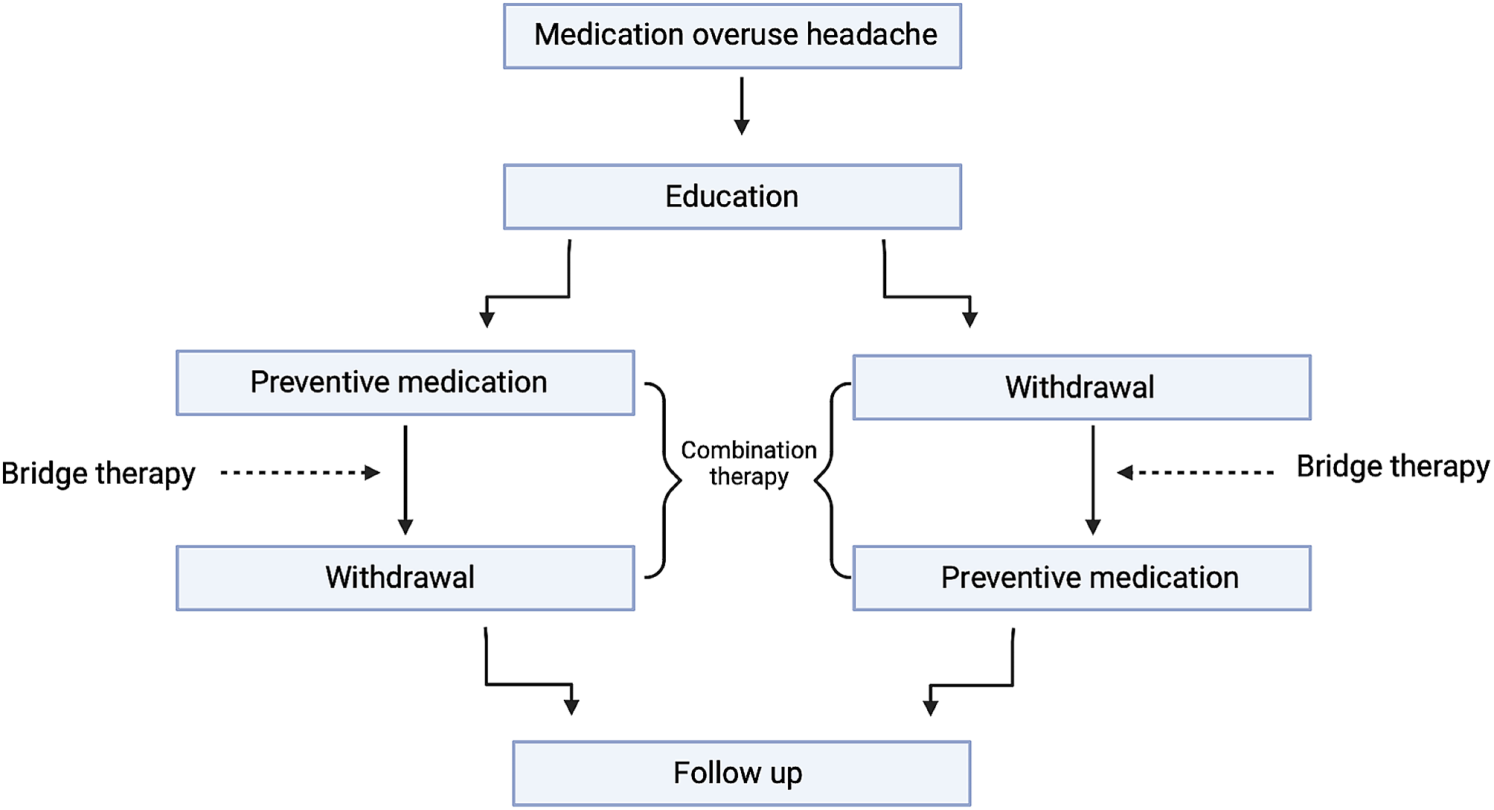
A flow chart displaying the possible treatment pathways for medication overuse headache

MOH therapy can be challenging for clinicians to manage. In some ways, providing treatment for something that has occurred, as a result of overuse of treatment creates a complicated barrier. Generally, there is no unanimity for the treatment of MOH, various clinics, countries and clinicians look to treat MOH in a personalised manner for each patient. Typically, there are two main routes adopted for MOH treatment (see Fig. 1).

A medium through which treatment would be eased is patient education. The first step to solving many headache-related issues is making the patient group aware of the issue and how some more adaptations to day-to-day habits can provide pain relief or facilitate improvement. In line with this, patient education is imperative. A study led by Fritsche and colleagues suggested that patients who were made aware of the prevention of MOH, through bibliotherapy resulted in no development of MOH in the study group and resulted in a significant reduction in both headache days and pain-related parameters [35]. Beyond clinical contact time, patients require an understanding of how to manage their headache and education has proved to be a successful method. This is largely consistent with our clinical experience, whereby once we have made the patient more aware of the problem, or informed them of it, issues in a follow – up appointment may be less. More recently, a group in Europe looked into the efficacy of mindfulness for patients with MOH. There has been some evidence for patients with migraine and mindfulness [36, 37] and exploring this for MOH led to some stimulating findings. The group conducted a phase-III single-blind randomised control trial (RCT) and investigated the efficacy of mindfulness as an extra treatment for patients with chronic migraine and MOH against a control group (treatment as usual). The primary endpoint was to attain a headache frequency reduction of ≥ 50% at the 12-month mark compared to baseline. The results showed that the addition of a mindfulness-based protocol to the existing treatment of chronic migraine related to MOH displayed superiority for the primary endpoint (78.4%), compared to the control group (48.3%). Furthermore, the mindfulness group had a ≥ 50% reduction in “headache” frequency, a reduction in pain intensity, quality of life and NSAIDs intake, compared to the control group [38].

### Medication withdrawal

Whether medication withdrawal following, or not, a change in headache prevention, or the direct change in prevention without medication withdrawal, is the best approach for these patients has been and still is a matter of debate.

The largest study trying to answer this question specifically was the COMOESTAS study [39]. This study included 694 patients and was conducted in European and Latin American countries. The combination of detoxification and preventive treatment was offered to 83% of patients. Forty-seven per cent went back to an episodic headache, meaning less than 15 headache days a month and up to 62% stopped overusing. However, the drop-out rate was as high as 30%, and 6% relapsed in a 6-month follow-up [40].

Of note, in a Danish study abrupt detoxification has been shown more feasible for patients and proven to be more effective in reducing headache-related anxiety than gradual medication withdrawal [41] as well as reducing disability as per the Headache Under-Response to Treatment index (HURT). Both programs led to improved estimated quality of life [42].

In the 1990s, medication withdrawal of ergotamine or other analgesics showed benefits even 5 years after the intervention although 39.5% recurred [43].

The main drawback of abrupt medication withdrawal is an expected initial worsening of the headache although this is drug-dependent [10]. It could be argued that inducing suffering is not an ideal approach to the problem. Moreover, such approaches have variable traction depending on the cultural context. Namely, triptan-withdrawal may be the shortest lasting, leading to a headache worsening for 4 days on average while opioid withdrawal symptoms can last for 10 days and some other clonidine may need to be used to ameliorate withdrawal symptoms [44].

### Bridge therapies

Although studies show the utility of medication withdrawal, few studies have been completed on the use of bridge therapies often used by neurologists before or following medication withdrawal.

Indeed, greater occipital nerve injections can certainly be used in this context and are known to be efficacious, safe and well-tolerated. Injection of local anesthetic and corticosteroids in the region of this peripheral nerve seems of particular utility given its projections to the trigeminal cervical complex. Its use has not been tested in a placebo-controlled trial in MOH patients. A recent meta-analysis on its use in cluster headache exemplifies the heterogeneity of the techniques utilised, the combination or not of corticosteroids and local anesthetics and the laterality of injections [45]. Our practice is to use 2 mL of lidocaine 2% along with 2 mL of methylprednisolone (80 mg). Studies with high-quality evidence are warranted.

Oral corticosteroids have not shown superiority when tested against placebo [46]. Intravenous dihydroergotamine (DHE) administered in infusions every 8 h for 5 days up to 11.25 mg has shown utility as an adjunctive therapy following withdrawal and treating the withdrawal headache [47, 48].

Repeated use may be helpful in refractory headaches that require withdrawal and long-term benefits have been reported [49]. The main predictor of poor outcome of DHE is the development of nausea during admission [48]. Protocols including potent antiemetic drugs, such as aprepitant, can tackle this side effect improving the outcomes [50].

Other treatments have been used as bridge therapies with only small studies and not as extended use. Among these, valproate was found positive in a study on chronic headache patients with and without medication overuse. Many of the patients included had previously failed IV DHE for the same purpose [51].

### Preventive therapy

Although this question had not been answered until recently, adding a preventive medication to complete withdrawal treatment may be of use given current evidence. Clinical trial-based evidence and clinically exhibited variations suggest that preventive therapy for the treatment of MOH holds value. Calcitonin gene-related peptide (CGRP) has an established position as a preventive of some primary headache disorders [52]. Emerging evidence has detailed that CGRP monoclonal antibodies are efficacious as a preventive treatment method for MOH. Dodick and colleagues conducted a subgroup analysis of three trials of galcanezumab: EVOLVE-1 and EVOLVE-2 for episodic migraine patients and REGAIN for chronic migraine patients. All trials studied 120 mg and 240 mg of galcanezumab, and the results indicated that compared to placebo, there was a significant fall in monthly migraine days. Furthermore, galcanezumab demonstrated a reduction in monthly medication overuse (MO) rates compared to placebo [53]. Moreover, in a study of participants treated with fremanezumab the treated group showed a 50% or greater reduction in headache days, compared to a placebo. Interestingly, as treatment went on some patients no longer exhibited MO, compared to placebo, over a 12-week period. More than headache characteristics and symptomology, patients treated with fremanezumab detailed better and improved scores for quality-of-life assessment, through the HIT-6 and MSQoL testing [54]. This study showed promising evidence for the treatment of MOH with another CGRP monoclonal antibody. Furthermore, a double-blind, randomised, placebo-controlled, phase 3 study compared 100 mg eptinezumab, 300 mg or placebo in participants with MOH. The results indicated that patients with CM and MOH treated with eptinezumab displayed a reduction in monthly migraine days compared to placebo. The results from this trial further encourage the use of CGRP monoclonal antibodies for the treatment of MOH [55]. Moving from CGRP-based evidence to preventive and withdrawal combination therapy, two Danish trials reported that a hybrid method of preventive treatment and withdrawal was more efficacious in the treatment of MOH than stand-alone methods of preventive-only or withdrawal-only. After 6 months, the primary outcome was a change in headache days per month. The results indicated that combination therapy: preventive and withdrawal was superior in the reduction of monthly headache days (reduction of 12.3) when compared to preventive alone (reduction of 9.9) or withdrawal alone (reduction of 8.5). Whilst all three methods were successful treatment forms, combination therapy yielded the greatest decline in headache days per month, over a 6-month course [56]. To confirm the findings over 6 months, Carlsen and colleagues further investigated the three approaches, over 1 year. The results showed no significant difference between the three forms of treatment. All three methods led to a reduction in monthly headache days, 10.8, 10.3 and 7.9 in the preventive plus potential: withdrawal at 6 months, withdrawal plus preventive and withdrawal with delayed potential preventive group, respectively. Overall, the authors concluded that based on the fastest effect of the treatment, withdrawal and early prevention was the most efficacious method. Schwedt and colleagues, in the United States, conducted an open-label, pragmatic clinical trial in which patients were randomised to either: preventive medication plus switching the overused medication to that of an alternative class (restricted to two days per week) or preventive medication plus the established overused medication [57]. In contrast to the Danish study, they concluded that the preventive medication plus switching the overused medication to that of an alternative class (restricted to days per week) is not superior to the alternative. They also detailed that during the first two weeks, both studied groups delivered comparable outcomes in terms of headache days data. Whilst both regions conducted slightly varying trials, the overall message is that, whether stand-alone or combined, the use of preventive therapy for the treatment of MOH is a reasonable approach. Other preventive medications previously reported as efficacious for the treatment of MOH in double-blind randomized controlled trials are onabotulinumtoxinA [58, 59] and topiramate [60–64]. Additionally, valproic acid [65], cannabinoids [66], pregabalin [64] and non-pharmacological treatments, such as acupuncture [67] and occipital nerve blockade have also [68], been studied; they are not considered routine approaches for patients with MOH.

### Some consensus

So, what is the best method for the therapy of MOH? There is not a simple answer to this. Depending on an array of things, such as previous dependence on medication overuse, existing co-morbidities, lifestyle, cultural context and symptomology, a personalised treatment plan is created, working in conjunction with the patient. Various guidelines are available, such as those of the European Academy of Neurology [69] and French Society of Neurology [70] guidelines have some alignment: they both suggest that education and then withdrawal is the best approach. On the contrary, an expert group panel joint guideline from the German Society of Neurology (DGN) and the German Migraine and Headache Society (DMKG) published a set of guidelines in which, clinicians and scientists amalgamated their expertise and experience to suggest that education should be a primary step, succeeding preventive therapy and then if this were to be insufficient, to then proceed to withdrawal [71] Table 2.

**Table 2:** Pros and cons of preventive and withdrawal treatment for medication overuse headache

	Preventive treatment	Withdrawal
Pros	• Recent evidence shows the efficacy of CGRP-antibodies in this subgroup. • Patients may be familiar with preventive therapies or have successful history of taking them previously.	• Treating what is believed to be the cause of the headache worsening seems reasonable. • A reduction in long-term medication burden and dependence.
Cons	• The question of whether withdrawal on its own could have led to the same improvement remains open. • Some preventive treatments may have side effects, and discovering what suits the patient may be challenging amidst an already existing issue.	• Many patients could relapse if the focus is not put on treating the underlying primary headache biology. • The initial abrupt abolition of medication can lead to initial discomfort and possible worsening of headache symptomology, which can make patients resort back to their original medication.

### MOH: caveats

There are some caveats, however, to the concept of MOH and it is noteworthy for us to consider these. One issue is whether MOH inevitably occurs in some patients, due to previous use of painkillers, for example, due to pre-existing medical conditions, aside from use as pain relief for headache. Alternatively, is it that an accumulation of overuse of medication which worsens further, an existing pre-morbid condition such as headache.

1. Types of medications: MOH can occur as a result or rather overuse of certain types of medication, whether they are over-the-counter drugs such as ibuprofen, which is less clear, or paracetamol or opioids. Detecting exactly what medication is contributing to the overuse is crucial, as thereon a plan can be determined. To further this, patients may be taking various medications that could contribute to medication overuse headache and so, determining what medication has attributed to MOH can be complex. Therefore, one possible challenge is the clinician-patient working together to identify what medication is the cause of the MOH.
2. Relapse risk: a study led by Sances and colleagues reported that around 22% of patients had relapsed into overuse after one year of withdrawal [72]. There have been varying possible reasons for relapse. Sances and colleagues suggested that given the effect of psychoactive substances such as alcohol and tobacco, patients tend to substance abuse. Given the addictive traits, such patients may be more susceptible to relapse, generally. To perhaps prevent relapse, it is important to understand the population of patients that may have a history or are currently under the influence of alcohol, illicit drugs or tobacco. This gives the clinician an insight into the general habits of the patient, and steers towards a better treatment method, to prevent relapse. To work in conjunction with a possible existing problem of substance abuse, it may be best to advise patients to withdraw from other substances, as working in tandem with withdrawal of medication overuse, the results may generate a better reduction in headache frequency and headache symptomology.
3. Study-related issues: a large caveat of MOH, is the subject-related information. Many patients, albeit understandably cannot recall specific medication consumption, dosage or time of use. Therefore, creating a database with patient data can be difficult and may not always be a true representative and questions. The external validity of research in MOH data may be questionable. A possible method to avoid inaccurate data being compiled and ambiguities of misdiagnosis, is a standardised international questionnaire battery for MOH diagnosis. This method would ensure that patients globally are asked the same questions and have a point of reference to ensure uniformity in data and therefore make apt comparisons and use of data. The clinical definition of MOH has evolved in the ICHD-3 [11], it is important we all use the same measures, particularly the same definition. For example, we ask patients questions about medication usage, headache history and quality, headache associations, cranial autonomic symptoms, premonitory/postdrome and triggers.
4. Geographical location: as established, MOH is treated differently in various regions, globally. The method of treatment and treatment plan depends upon patient presentation in a specific region, cultural considerations and resource availability. In some countries, it may be that simple analgesics or triptans are contributing to MOH, whereas, in another country, it is opioids and barbiturates. The difference in cause, or at least association, of MOH will result in variations in care, and so data interpretation should be done with knowledge of what could have caused MOH and how cases present in any clinical setting.
5. Possible misdiagnosis: there is a possibility that misdiagnosis of MOH may occur. It is an uncomfortable situation to explain to the patient about their misuse or overuse of medication, as this may not always be the case. Moreover, by possible misdiagnosis, clinicians risk diverting the problem and missing a more important aspect of the headache diagnosis such as a secondary cause or worsening of a primary headache disorder.

## Conclusion

Through an analysis of the available literature, our clinical experience and patient interactions, we provide an overview of MOH, highlighting some caveats to consider. Whilst the evidence indicates that MOH is prevalent amongst primary headache disorder patients, there is scope for better diagnosis, disorder management, patient education and clinical understanding. As we surmise that MOH is not a stand-alone issue, but rather developed, it is important for future studies to be conducted over an extended period. Longitudinal studies ensure changes and developments of the headache are from medication overuse to treatment of an existing headache, rather than from a previous condition. Furthermore, pharmacological treatments are not adequate for the treatment of some patients MOH. A combination of many aspects allows mitigation of this burden and working in conjunction with patients and clinicians globally will enable treatment of the problem. Overall, the evidence suggests that there should be an emphasis on patient education. In-depth medical history taking and informed clinical decisions to ensure that MOH is a strictly, classified entity, are both important. As a community, there is room for improvement, especially when designing studies and clarifying definitions, patient data and clinician understanding must be refined to create a more succinct treatment environment.

## Data Availability

No datasets were generated or analysed during the current study.

## Abbreviations

MOH: Medication-overuse headache
ICHD-3: International classification of headache disorders-3
FDG-PET: ^18^F-Fluorodeoxyglucose - positron emission tomography
fMRI: Functional magnetic resonance imaging
PAG: Periaqueductal grey
CGRP: Calcitonin gene-related peptide
MO: Medication overuse
DHE: Dihydroergotamine
MRI: Magnetic resonance imaging
NSAID: Non-steroidal anti-inflammatory drugs
DGN: German Society of Neurology
DMKG: German Migraine and Headache Society
